# Macrophage-directed T cell recruitment augments IFN-Mediated suppression of cardiac reprogramming *in vivo*

**DOI:** 10.1093/lifemedi/lnag005

**Published:** 2026-04-07

**Authors:** Yihong Cai, Hao Wang, Junbo Yang, Qianhe Li, Yuxiang Dai, Yang Zhao

**Affiliations:** State Key Laboratory of Natural and Biomimetic Drugs, Ministry of Education Key Laboratory of Cell Proliferation and Differentiation, Beijing Advanced Center of Cellular Homeostasis and Aging-Related Diseases, Institute of Advanced Clinical Medicine, Center for Life Sciences, College of Future Technology, Peking University, Beijing 100871, China; State Key Laboratory of Natural and Biomimetic Drugs, Ministry of Education Key Laboratory of Cell Proliferation and Differentiation, Beijing Advanced Center of Cellular Homeostasis and Aging-Related Diseases, Institute of Advanced Clinical Medicine, Center for Life Sciences, College of Future Technology, Peking University, Beijing 100871, China; Peking-Tsinghua Center for Life Science, Academy for Advanced Interdisciplinary Studies, Peking University, Beijing 100871, China; State Key Laboratory of Natural and Biomimetic Drugs, Ministry of Education Key Laboratory of Cell Proliferation and Differentiation, Beijing Advanced Center of Cellular Homeostasis and Aging-Related Diseases, Institute of Advanced Clinical Medicine, Center for Life Sciences, College of Future Technology, Peking University, Beijing 100871, China; Department of Cardiology, Zhongshan Hospital, Fudan University; Shanghai Institute of Cardiovascular Diseases, Shanghai 200032, China; State Key Laboratory of Cardiovascular Diseases, Zhongshan Hospital, Fudan University, Shanghai 200032, China; National Clinical Research Center for Interventional Medicine, Shanghai 200032, China; Department of Cardiology, Zhongshan Hospital, Fudan University; Shanghai Institute of Cardiovascular Diseases, Shanghai 200032, China; State Key Laboratory of Cardiovascular Diseases, Zhongshan Hospital, Fudan University, Shanghai 200032, China; National Clinical Research Center for Interventional Medicine, Shanghai 200032, China; State Key Laboratory of Natural and Biomimetic Drugs, Ministry of Education Key Laboratory of Cell Proliferation and Differentiation, Beijing Advanced Center of Cellular Homeostasis and Aging-Related Diseases, Institute of Advanced Clinical Medicine, Center for Life Sciences, College of Future Technology, Peking University, Beijing 100871, China; Peking-Tsinghua Center for Life Science, Academy for Advanced Interdisciplinary Studies, Peking University, Beijing 100871, China

**Dear editor,** 

Myocardial infarction (MI) poses significant challenges for global health. Direct conversion of cardiac fibroblasts (CFs) to cardiomyocytes (CMs) *in vivo* via overexpression of cardiac transcription factors MGT (*Mef2c*, *Gata4*, and *Tbx5*) holds great potential for cardiac regeneration. After MI, heart repair proceeds with an inflammation stage characterized by monocyte infiltration into the infarct zone, which establishes an immune microenvironment. Our previous study has shown that macrophages function as a crucial microenvironmental cell type that suppresses *in vivo* production of induced cardiomyocyte-like cells (iCMs) through IFN-β–IFNAR–pSTAT1–CCL2/7/12 axis in a self-reinforcing manner mediated by both CFs and macrophages [1]. We also demonstrated that disruption of this loop by knockdown of *Ifnar2*, which encodes Ifnar2, a subunit of the type I interferon receptor and is critical for IFN-β signaling, significantly enhanced the efficiency of iCM induction [1].

Macrophages are not the only immune cell type in the post-myocardial infarction microenvironment. Actually, both macrophages and T cells accumulated rapidly following myocardial infarction [2, 3]. In the microenvironment of the infarct region, recruited macrophages in the injured heart can further recruit CXCR3^+^ T cells by secreting CXCL10 in injured heart [4]. T cells, particularly T helper 1 (Th1) cells, and CD8^+^ T cells enriched in the infarct region [3], also secrete IFN-γ. This cytokine may also activate STAT1 phosphorylation in CFs through the IFNGR receptor [5, 6], sharing a common downstream signaling pathway with IFN-β. Thus, it is intriguing what the contribution of T cells is in regulating *in vivo* direct cardiac reprogramming. Based on our previous findings, STAT1 phosphorylation suppresses cardiac reprogramming [1].

Consistently, by Western blot, we found STAT1 phosphorylation level was indeed increased in CFs isolated from adult mice with myocardial infarction (MICFs) after exogenous IFN-γ treatment (Fig. 1A). Reanalysis of a public single-cell RNA sequencing dataset (GSE120064) from injured mouse hearts revealed that *Cxcl10* expression exceeded that of other CXCR3 ligands (e.g., *Cxcl9*, *Cxcl11*) and was highest in macrophages among all cell types post-injury. (Figs. 1B, S1A and S1B). In agreement with previous studies, transwell migration assays showed that CXCL10 could induce significant migration of CD8^+^ cytotoxic T lymphocytes (CTLs) from OT-I mice in a concentration-dependent manner, and CXCL10 knockout in macrophage reduced the ratio of macrophage-mediated CTL recruitment by about 1.5-fold compared to the sgRNA non-targeting controls (Figs.1C and S1C).

**Figure 1.**
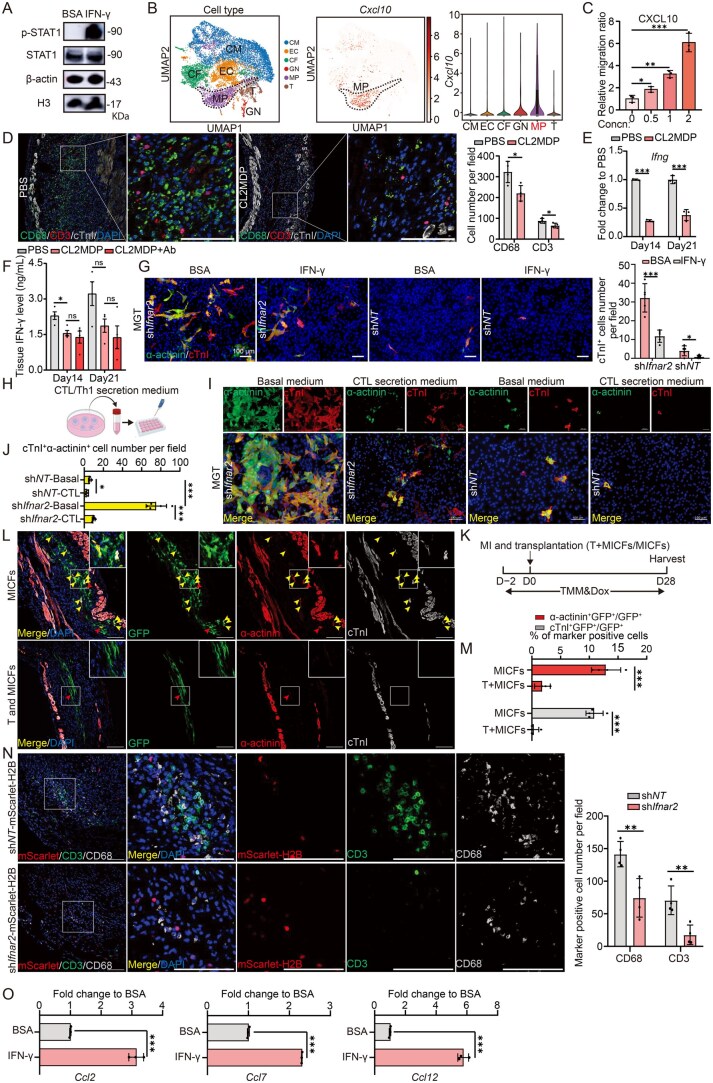
Macrophages recruit T cells that secrete IFN-γ to suppress cardiac reprogramming.(A) Western blot analyses for p-STAT1 expression in MICFs expressing MGT and treated with 100 ng/mL IFN-γ or BSA for 7 days. (B) sc-RNA seq analysis of *Cxcl10* expression for each cell type. (C) Quantification of migration ratio from the T cells (cytotoxic T lymphocytes, CTLs) transwell migration assay with 0–2 μg/mL CXCL10 chemokines (*n* = 3). (D) Representative IF images for CD3 and CD68 in D7 MI hearts treated with CL2MDP or PBS (left panel), with quantification of the absolute number (right panel). (E) RT-PCR analyses for *Ifng* expression in MI hearts treated with CL2MDP or PBS at day 14 and day 21 (*n* = 3). (F) ELISA assay for IFN-γ level in MI hearts treated with PBS, CL2MDP, or CL2MDP with anti-CD8 and anti-CXCR3 antibodies at day 14 and day 21 (*n* = 4). (G) Representative IF images for cTnI on MGT+sh*Ifnar2* or sh*NT* transduced MICFs treated with 3 ng/mL IFN-γ or BSA (left panel), with quantification of the absolute number in (right panel) (*n* = 5). (H) Schematic diagram of T-cell-conditioned medium. (I and J) Representative IF images for α-actinin and cTnI on MGT+sh*Ifnar2* or sh*NT* transduced MICFs treated with T cell conditional medium or basal medium (I), with quantification of the absolute number in (J) (*n* = 5). (K–M) Schematic diagram and timeline of MICFs and T cells co-transplantation into WT mouse hearts (K). Representative IF images for α-actinin, cTnI, and GFP on co-transplantation of T cells and MICFs expressing MGT and sh*Ifnar2* into MI hearts 4 weeks (L), with quantification of the percentage in (M) (*n* = 4). (N) Representative IF images for CD68, CD3, and mScarlet-H2B from *in vivo* MICFs knockdown experiment (left panel), with quantification of the absolute number (right panel) (*n* = 4). (O) qPCR analysis of the *Ccl2/7/12* expression at 3 days after treatment of 100 ng/mL IFN-γ or BSA in MICFs (*n* = 3). All data are presented as the means ± SD. The one-way ANOVA followed by Dunnett’s multiple comparisons test (C, F), two-way ANOVA followed by Tukey’s multiple comparisons test (J) or unpaired *t*-test (E, G, M, N, and O) were used to determine the significance of differences between two groups. NS, not significant, *P < 0.05, **P < 0.01, ***P < 0.001, Scale bars, 100 μm.

We subsequently depleted macrophages by injecting macrophage scavenger, clodronate (CL2MDP) packaged in liposomes to specifically inhibit macrophage infiltration [7] and quantified CD3^+^ T cells at 1 weeks after MI to investigate whether macrophages played a role in T cell recruitment after cardiac injury. Consistent with previous reports, Immunofluorescence (IF) staining indicated that CD68^+^ macrophages and CD3^+^ T cell numbers were lower in the macrophage-depleted group than that in the PBS group (Fig. 1D). In addition, qPCR assay revealed that IFN-γ expression was decreased by about 3.5-fold at 2 weeks post MI and decreased by about 2.6-fold at 3 weeks post MI in the macrophage-depleted group compared with the PBS group (Fig. 1E). Similarly, ELISA assays demonstrated that tissue IFN-γ levels were reduced by about 1.5-fold at 2 weeks post-MI and by about 1.7-fold at 3 weeks post-MI in the macrophage-depleted group compared to the PBS-treated ­controls. However, further inhibition of CTLs and Th1 cells infiltration into infarct region using combined anti-CD8 and anti-CXCR3 antibody treatment did not significantly reduce IFN-γ levels beyond those observed in macrophage-depleted mice (Fig. 1F). These results indicated that the recruitment of T cells is impaired with a reduction in the number of infiltrating macrophages following myocardial infarction.

To further investigate whether T cells and IFN-γ can suppress cardiac reprogramming, we added IFN-γ to the cardiac reprogramming medium at concentrations equivalent to those *in vivo*. MGT+sh*Ifnar2* and sh*NT* were utilized as basal reprogramming factor combinations, respectively. After 4 weeks, the number of cTnI^+^ (cardiac troponin I, also known as Tnni3) cells significantly decreased by about 2.7-fold in the MGT+sh*Ifnar2* group and by about 5.0-fold in the MGT+sh*NT* group upon the addition of IFN-γ (Fig. 1G). We then cultured CTLs and Th1 cells *in vitro*, harvesting supernatants after 4 days and 5 days of ­culture, respectively (Fig. 1H), which were subsequently added to the culture medium of MICFs transduced with MGT+sh*NT* or MGT+sh*Ifnar2*. ELISA assay confirmed the presence of IFN-γ in each type of conditional medium, with concentrations of 1.8 ng/mL in CTL conditional medium and 146.1 ng/mL in Th1 conditional medium (Fig. S1D and S1E). After 4 weeks cultured with CTL conditional medium, IF analysis revealed a significant decrease in the numbers of cTnI^+^α-actinin^+^ (cardiac sarcomere protein, encoded by gene *Actn2*) cells in comparison to untreated controls, by about 1.9-fold in MGT+sh*NT* groups and about 7.7-fold in MGT+sh*Ifnar2* groups, respectively (Fig. 1I and 1J). Similarly, the number of cTnI^+^α-actinin^+^ decreased significantly with Th1 conditional medium in comparison to untreated controls, by about 8.5-fold in MGT+sh*NT* groups and about 12.8-fold in MGT+sh*Ifnar2* groups, respectively (Fig. S1F and S1G). These results indicated that T cells can suppress cardiac reprogramming independent of type I Interferon receptors.

Furthermore, we neutralized IFN-γ activity in conditional medium by adding an IFN-γ-blocking antibody. cTnI^+^α-actinin^+^ cell numbers increased upon IFN-γ neutralization in Th1 conditional medium compared to untreated controls, with an increase of about 6.4-fold in MGT+sh*NT* groups and about 9.6-fold in MGT+sh*Ifnar2* groups, respectively (Fig. S1F and S1G). Similarly, the number of cTnI^+^α-actinin^+^ increased upon IFN-γ neutralization in CTL conditional medium compared to untreated controls, by about 4.8-fold in MGT+sh*NT* groups and about 2.8-fold in MGT+sh*Ifnar2* groups, respectively (Fig. S1H and S1I). In summary, these results demonstrated that IFN-γ secreted by T cells can impede CF reprogramming into CMs, and that blocking extracellular IFN-γ signaling can restore transdifferentiation efficiency.

To investigate the regulation of T cells on cardiac reprogramming *in vivo*, we infected MICFs with lentiviruses expressing MGT+sh*Ifnar2* and transplanted the *in vitro* infected cells with or without T cells into the hearts of mice after MI (Fig. 1K). After 4 weeks of transplantation, IF staining of cryo-sections showed that approximately 12.9% of eGFP^+^ cells were α-actinin^+^ and about 10.9% were cTnI^+^ in the MICF only group. However, only about 1.8% α-actinin^+^ and 0.4% cTnI^+^ were observed in the “MICF plus T cells” co-transplantation group (Fig. 1L and 1M). Taken together, these results suggest that T cells play an inhibitory role in cardiac reprogramming *in vitro* and *in vivo*.

Given that either macrophages or T cells inhibit *in vivo* cardiac reprogramming, it is crucial to evaluate the relationships between the two cell types. According to our previous study [1], we found that MICFs communicated with macrophages through a positive feedback loop and led to T cell recruitment by macrophages. In light of these finding, we sought to investigate whether there is a more comprehensive loop among MICFs, macrophage and T cells, and whether solely *Ifnar2* KD can disrupt that loop. After 2 weeks, IF staining of MI heart sections showed that the numbers of macrophages decreased by about 1.9-fold and T cells decreased by about 4.0-fold in the vicinity of MICFs in sh*Ifnar2* group, compared with sh*NT* group (Fig. 1N). Additionally, qPCR assays further indicated that IFN-γ treatment also led to higher expression levels of *Ccl2*, *Ccl7*, and *Ccl12* chemokines in MICFs *in vitro* (Fig. 1O) via activation of STAT1 phosphorylation (Fig. 1A). Correspondingly, macrophages in injured myocardial tissue expressed the highest levels of *Ccr2*, the receptor for CCL2, CCL7, and CCL12, indicating that they are the primary cell type responding to these chemotactic signals (Fig. S1A and S1B).

Taken together, these findings suggested that MICFs recruit monocytes/macrophages after myocardial infarction, and macrophages further recruit T cells. Macrophage-derived IFN-β and T cell-secreted IFN-γ cooperatively phosphorylate STAT1 in MICFs, potentiating *Ccl2*/*Ccl7*/*Ccl12* expression and creating a consolidated self-stimulatory loop (Fig. 2). This tripartite cell-mediated IFN signaling circuit therefore presents a critical molecular barrier for *in situ* cardiac reprogramming after myocardial infarction. Eliminating this barrier unlocks cardiac regeneration potential through the simple MGT factors. Notably, macrophages are identified as the primary hindrances to *in situ* reprogramming, while T cells play a supportive role as a follower. Suppressing the recruitment of macrophages leads to a corresponding reduction in T cells, thereby minimizing both of their inhibitory effects. In conclusion, our findings elucidate a mechanism of immune-fibroblast cell communication that can exacerbate pathological processes and hinder cardiac reprogramming. This concept is further supported by recent studies [8, 9].

**Figure 2.**
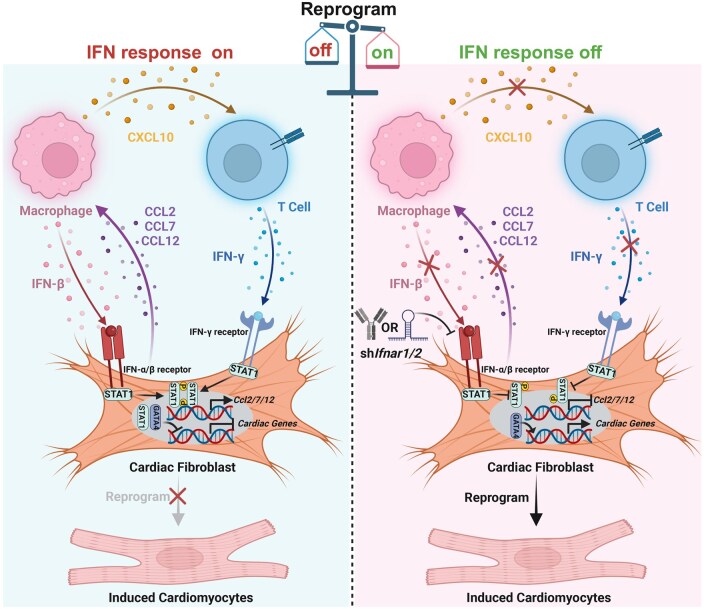
A self-stimulating positive feedback loop between MICFs, macrophages, and T cells. Schematic diagram of a self-stimulating positive feedback loop between MICFs, macrophages, and T cells.

## Research limitations

Although our study found that T cells and their secreted IFN-γ can inhibit the conversion of CFs into CMs, this does not exclude other potential roles of T cells in regulating reprogramming. For example, T cells can secrete other cytolytic factors, such as granzymes and perforin, in addition to IFN-γ. These points will be investigated in future studies.

## Supplementary Material

lnag005_Supplementary_Data

## Data Availability

Public scRNA-seq datasets supporting the findings of this study are available through the GEO SuperSeries access number GSE156728.
