# Supplementary material for: Macrophage-directed T cell recruitment augments IFN-Mediated suppression of cardiac reprogramming in vivo
Source: Life Med. 2026 Apr 7;5(1):lnag005. doi: 10.1093/lifemedi/lnag005 (PMC13131212; doi:10.1093/lifemedi/lnag005)
Supplement: lnag005_Supplementary_Data [file lnag005_supplementary_data.zip › Macrophage_SI.docx]

**Macrophage-Directed T Cell Recruitment Augments IFN-Mediated Suppression of Cardiac Reprogramming *In Vivo***

Yihong Cai^1,#^, Hao Wang^1,2,#^, Junbo Yang^1,#^, Qianhe Li^3,4,5,#^, Yuxiang Dai^3,4,5,*^, Yang Zhao^1,2,*^

^1^State Key Laboratory of Natural and Biomimetic Drugs, Ministry of Education Key Laboratory of Cell Proliferation and Differentiation, Beijing Advanced Center of Cellular Homeostasis and Aging-Related Diseases, Institute of Advanced Clinical Medicine, Center for Life Sciences, College of Future Technology, Peking University, Beijing 100871, China

^2^Peking-Tsinghua Center for Life Science, Academy for Advanced Interdisciplinary Studies, Peking University, Beijing 100871, China

^3^Department of Cardiology, Zhongshan Hospital, Fudan University; Shanghai Institute of Cardiovascular Diseases, Shanghai 200032, China

^4^State Key Laboratory of Cardiovascular Diseases, Zhongshan Hospital, Fudan University, Shanghai 200032, China

^5^National Clinical Research Center for Interventional Medicine, Shanghai 200032, China

^#^These authors contributed equally to this work.

^*^Correspondence: [yangzhao@pku.edu.cn](mailto:yangzhao@pku.edu.cn) (Y.Z.), dai.yuxiang@hotmail.com (Y.D.)

**Methods**

**Cardiac reprogramming *in vitro***

MICFs were infected with freshly prepared lentiviruses (5×10^6^ TU/mL) along with 8 µg/mL polybrene at approximately 90% confluence in 24-well plates. After 24 h, the culture medium of the MICFs was replaced with iCMs media (a 4:1 mixture of DMEM and M199, supplemented with 10% FBS, 10% KSR, 1% P/S, 1% NEAA, 1% Glutamax, 2 μM SB431542, and Baricitinib, along with 2 μg/mL doxycycline), with the medium being changed every two days.

For *in vitro* immunofluorescence, cells underwent fixation in 4% paraformaldehyde (15 min) followed by permeabilization in 0.1% Triton-X/PBS (10 min). Blocking was performed with 3% normal donkey serum (1 h, room temperature). Primary antibody incubation against cTnI (Abcam ab56357, 1:100) and α-actinin (Sigma A7311, 1:500) proceeded at 4℃ overnight. After PBS washes, samples were exposed to DAPI and secondary antibodies: anti-goat Alexa Fluor 555 (Invitrogen A32816, 1:1000) and anti-rabbit Alexa Fluor 488 (Invitrogen A32790, 1:1000) for 1 h at room temperature. Representative images were captured using an inverted fluorescence microscope (AXIO Vert.A1), and high-content whole-well images were acquired with the Cell Discoverer 7 system from Zeiss. For quantification, CellProfiler version 3.1.8 was used to perform a standard processing pipeline, including illumination correction, single-cell segmentation, and absolute cell counting based on marker gene expression in whole-well microscopy images.

**Cell transplantation**

MICFs, at approximately 90% confluence, were infected with lentivirus and harvested two days post-infection. A left thoracotomy was performed on C57BL/6 mice, and 10^6^ cultured cells were injected into the left ventricle immediately following left anterior descending ligation. In the co-transplantation experiments involving T cells and MICFs expressing MGT/eGFP/shRNA, 5 × 10^5^ cultured CTLs (Cytotoxic T cells) and 5 × 10^5^ MICFs were injected into the left ventricle. To reduce the immune response, mice received TMM treatment (2 mg/kg/day Tacrolimus, 20 mg/kg/day Methylprednisolone, and 20 mg/kg/day Mycophenolate mofetil) via intraperitoneal injection for two days prior to surgery. The TMM immunosuppressants were administered daily until harvest. Additionally, 2 mg/mL doxycycline with 5% sucrose was added to the drinking water to induce gene expression before surgery.

**Co-culture experiments**

Co-culture experiments with CD8^+^ T cells and MICFs were performed using mature cytotoxic T lymphocytes (CTLs) or T helper 1 (Th1) cells and concentrated fibroblast-conditioned medium. Cells isolated from OT-1 mice spleen were stimulated with OVA_257-264_ and 10 ng/mL IL-2 for 2 days, then collected cells and cultured in fresh medium with 10 ng/mL IL-2 for 2 days, after which most of cells had differentiated into CTLs^8^. After three days, the supernatant from the CTLs medium was collected and incorporated into the reprogramming medium, which was then added to MICFs expressing MGT and sh*Ifnar2/NT*.

Co-culture experiments of CD8^+^ T cells and MICFs were conducted using CTLs or Th1 cells with concentrated fibroblast-conditioned medium. For CTL conditional medium preparation, splenocytes from OT-1 mice were resuspended in RPMI 1640 supplemented with 10% FBS, 50 μM β-mercaptoethanol, and 1% penicillin/streptomycin (P/S), and 1×10^7^ cells were cultured in a 10 cm dish. These splenocytes were stimulated with OVA_257-264_ and 10 ng/mL IL-2 (MCE, HY-P7077) for 2 days, followed by centrifugation and culture in fresh medium with 10 ng/mL IL-2 for an additional 2 days. Most of the cells in the culture had differentiated into CTLs^8^. After 3 days, the CTL supernatant was collected, substituted for DMEM in the reprogramming medium described above to create CTL conditional medium, and applied to MICFs expressing MGT and sh*Ifnar2*/*NT*.

For Th1 conditional medium, 6-well plates were coated with 1 mL PBS containing 2 μg/mL CD3e antibody (Invitrogen, clone: 145-2C11) and incubated overnight at 4°C. CD4^+^ T cells, isolated from C57 mouse spleens using the MojoSort™ Mouse CD4 T Cell Isolation Kit (BioLegend, 480005), were resuspended (1.5×10^6^ cells in 500 μL) in Th1 differentiation medium, a mixture of RPMI1640 with 10% FBS, 1% NEAA, 1% P/S, 50 μM β-ME, 0.5 μg/mL anti-CD28 antibody (Invitrogen, clone: 37.51), 1 μg/mL anti-IL-4 antibody (Invitrogen, clone: 11B11), 5 ng/mL IL-2 (MCE, HY-P7077), and 10 ng/mL IL-12 (MCE, HY-P70666). After 4 days of differentiation, the Th1 supernatant was collected, replaced DMEM in the reprogramming medium to form Th1 conditional medium, and added to MICFs expressing MGT and sh*Ifnar2*/*NT*.

IFNγ in the conditional medium was neutralized using an IFN-γ antibody (Invitrogen, clone: XMG1.2) at a concentration of 40 ng/mL for every 1 ng/mL of IFN-γ.

**Tissue slice immunofluorescence**

Hearts were fixed in 0.4% paraformaldehyde for 4 hours and then dehydrated overnight in 30% sucrose. They were subsequently embedded in OCT (Macgene) for freezing in liquid nitrogen. The hearts were sectioned vertically into 10 µm slices. Sections were stained with primary antibodies against α-actinin (Sigma, #A7311, 1:500), cTnI (Abcam, #ab56357, 1:100), GFP (Abcam, #ab13970, 1:500), CD68 (Abcam, #ab125212, 1:500), and CD3 (Invitrogen, #14-0032-81, 1:100), followed by secondary antibodies conjugated with Alexa 488, 555, 647, and DAPI. All sections were imaged using an A1R confocal microscope (Nikon).

**Analysis of online scRNA-seq data**

Mouse heart single cell expression data analysis was available from Gene Expression Ominbus (GSE120064).

**RT-qPCR assay**

Total RNA from MICFs or heart samples was isolated using TRIzol (Thermo Fisher Scientific) and subsequently extracted with the Direct-Zol RNA Miniprep Kit (Zymo Research, R2062). For cDNA synthesis, 1 μg of RNA was reverse-transcribed with Oligo-dT (Vazyme, R333-01). qPCR was performed on a Real-Time PCR system (q225, Kubo Tech) using SYBR Green PCR Master Mix (Vazyme, Q321-03), and expression levels were normalized to Gapdh. The primers used are listed in Table S2 and S3.

**ELISA**

For cardiac tissue samples, myocardial infarction was induced in C57 mice via permanent ligation of the left anterior descending coronary artery. On postoperative day 5, mice received intravenous tail vein injection of 200 μL CL2MDP (Yeasen, 40337ES08) to deplete macrophages, combined with intraperitoneal injection of anti-CD8α antibody (Selleck, clone 2.43) and anti-CXCR3 antibody (Selleck, clone CXCR3-173) with 200 μg per mouse every 4 days to deplete CTL/Th1 cells. Cardiac tissues from infarct and border zones were harvested on days 14 and 21 post-MI, rinsed with PBS, and weighed. Samples were minced and homogenized on ice in PBS containing protease inhibitors at a 1:1 (*w*/*v*) ratio. Homogenates were centrifuged at 5000 *g* for 5–10 min at 4°C, and supernatants were collected for analysis.

For Th1 and CTL conditional medium, prepared culture medium were directly subjected to analysis.

IFN-γ levels were measured using an ELISA kit (Elabscience, E-EL-M0048). Briefly, blank wells, standard curve wells, and sample wells (all in technical replicates) were loaded with assay diluent, recombinant standards, or diluted samples, respectively. Plates were incubated at 37℃ for 90 min, followed by one hour incubation with biotinylated detection antibody. After 3 washes, HRP-conjugated streptavidin was added for 30 min. Following 5 washes, TMB substrate was added and incubated for 15 min at 37°C under light-protected conditions. The reaction was terminated with stop solution, and optical density was immediately measured at 450 nm. IFN-γ concentrations were interpolated from the standard curve.

**Quantification and statistical analysis**

All experimental data were presented as the mean ± SD. “*n*” represented the number of animals or samples and was indicated in the figure legends. For statistical evaluation, unpaired Student’s *t*-test or One-way ANOVA followed by the Dunnett’s multiple comparisons test (to a single control group) or the Tukey’s multiple comparisons test (among groups) were used to determine the difference between groups. For multiple group comparisons with >2 variables the Two-way ANOVA followed by Tukey’s multiple comparisons test was performed using Graphpad Prism software, as indicated in figure legends. Differences with *P* values < 0.05 were regarded as significant.

**Ethics approval**

The animal experiments complied with regulations of the Institutional Animal Care and Use Committee of Peking University (Animal use license number: IMM-ZhaoY-2).

**Table S1. Sequence of shRNA and sgRNA (related to methods)**

| **Gene** | **Forward sequence& reverse sequence** | **Target sequence** |
| --- | --- | --- |
| sh*Ifnar2* | CCGGGGGAGAGAAAGGGAAAGAACTCGAGTTCTTTCCCTTTCTCTCCCTTTTTG  AATTCAAAAAGGGAGAGAAAGGGAAAGAACTCGAGTTCTTTCCCTTTCTCTCCC | GGGAGAGAAAGGGAAAGAA |
| sh*NT* | CCGGCCTAAGGTTAAGTCGCCCTCGCTCGAGCGAGGGCGACTTAACCTTAGGTTTTTG  AATTCAAAAACCTAAGGTTAAGTCGCCCTCGCTCGAGCGAGGGCGACTTAACCTTAGG | CCTAAGGTTAAGTCGCCCTCG |

**Table S2. Primer sequence of qPCR (related to methods)**

| **Gene** | **Forward** | **Reverse** |
| --- | --- | --- |
| *Ccl2* | TTAAAAACCTGGATCGGAACCAA | GCATTAGCTTCAGATTTACGGGT |
| *Ccl7* | GCTGCTTTCAGCATCCAAGTG | CCAGGGACACCGACTACTG |
| *Ccl12* | ATTTCCACACTTCTATGCCTCCT | ATCCAGTATGGTCCTGAAGATCA |
| *Ifng* | ATGAACGCTACACACTGCATC | CCATCCTTTTGCCAGTTCCTC |
| *Gapdh* | AGGTCGGTGTGAACGGATTTG | TGTAGACCATGTAGTTGAGGTCA |

**Table S3. Mouse genotyping primer sequences (related to methods)**

| **Mouse line** | **Forward** | **Reverse** |
| --- | --- | --- |
| *OT-1* | CAGCAGCAGGTGAGACAAAGT | GGCTTTATAATTAGCTTGGTCC |
