# Supplementary material for: Macrophage-directed T cell recruitment augments IFN-Mediated suppression of cardiac reprogramming in vivo
Source: Life Med. 2026 Apr 7;5(1):lnag005. doi: 10.1093/lifemedi/lnag005 (PMC13131212; doi:10.1093/lifemedi/lnag005)
Supplement: lnag005_Supplementary_Data [file lnag005_supplementary_data.zip › Supplementary Data_0711_作者返回2.pdf]

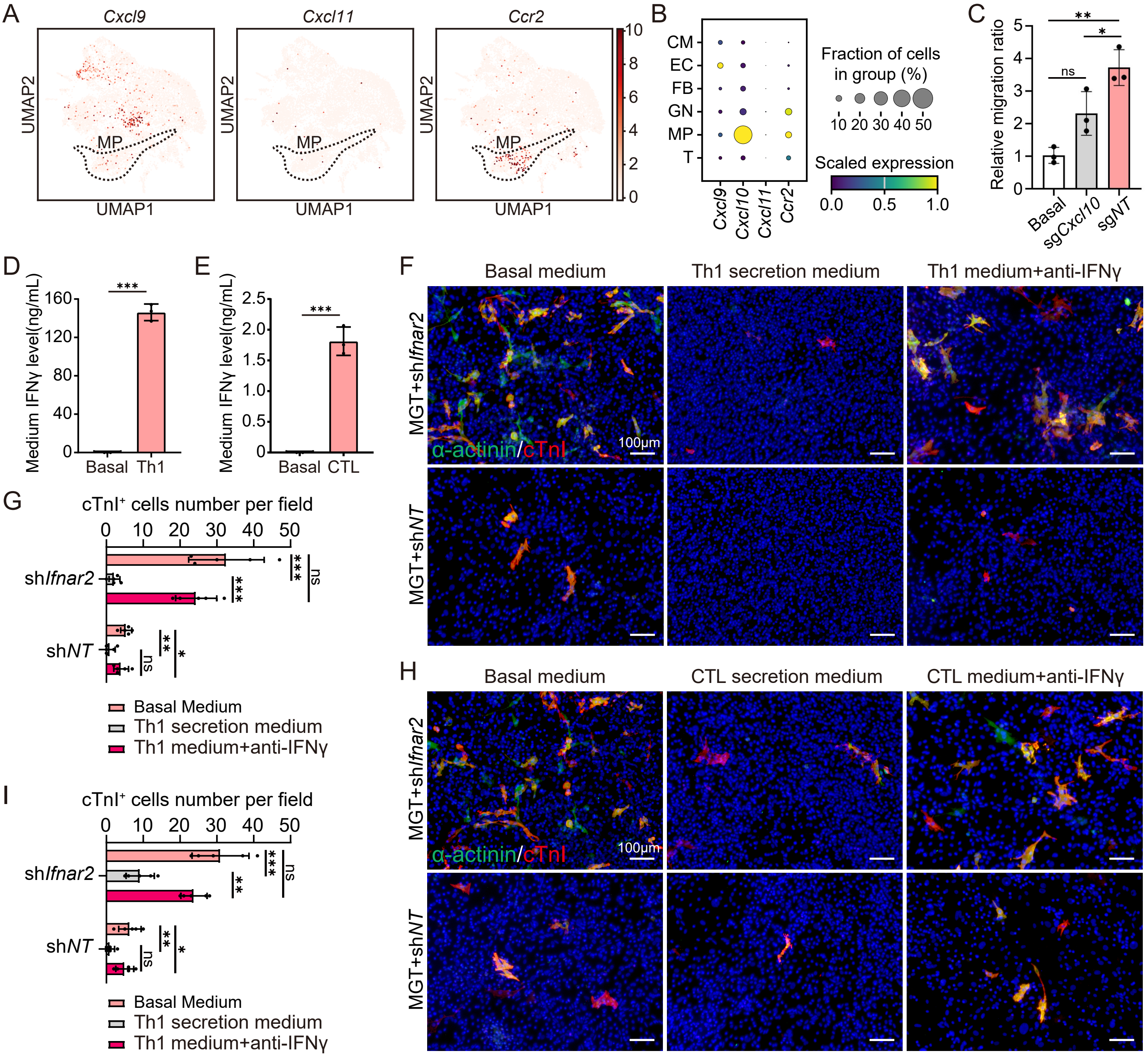

Supplementary Figure 1. (**A** and **B**) Sc-RNA seq analysis of Cxcl9, Cxcl11, Ccr2 expression for each cell types, shown in UMAP plot (A) and dot plot (B). (**C**) Quantification of migration ratio from the T cells transwell migration assay, sgNT- or sgCxcl10- infected macrophages or basal medium were loaded into the lower chamber, *n* = 3. (**D** and **E**) ELISA assay for IFN-γ level in Th1 (D) and CTL (E) conditionalmedium. n=3. (**F** and **G**) Representative IF images for α-actinin (green) and cTnI (red) on MGT+shIfnar2 or shNT transduced MICFs treated with Th1 conditional medium, basal medium or neutralized IFN-γ by added IFN-γ antibody (F), with quantification of the absolute number in (G). *n* = 5. (**H** and **I**) Representative IF images for α-actinin (green) and cTnI (red) on MGT +shIf-nar2 or shNT transduced MICFs treated with CTL conditional medium, basal medium or neutralized IFN-γ by added IFN-γ antibody (H), with quantification of the absolute number in (I). *n* = 5.

Data is presented as the means±SD. The one-way ANOVA followed by Tukey's multiple comparisons test(C, G, I), unpaired *t*-test (D, E) were used to determine the significance of differences between two groups. NS, not significant, $*p < 0.05$, $**p < 0.01$, $**p < 0.001$.
